# Supplementary material for: Compensating for visibility artefacts in photoacoustic imaging with a deep learning approach providing prediction uncertainties
Source: Photoacoustics. 2020 Oct 27;21:100218. doi: 10.1016/j.pacs.2020.100218 (PMC7750172; doi:10.1016/j.pacs.2020.100218)
Supplement: MMC S1 [file mmc1.pdf]

Compensating for visibility artefacts in photoacoustic imaging with a deep learning approach providing prediction uncertainties: supplementary materials

Guillaume Godefroy[a,*], Bastien Arnal[a], Emmanuel Bossy[a]

[a] *Univ. Grenoble Alpes, CNRS, LIPhy, 140 rue de la Physique, CS 40700, 38058 Grenoble CEDEX 9, FRANCE*

Abstract

This document provides supplementary information to "Compensating for visibility artefacts in photoacoustic imaging with a deep learning approach providing prediction uncertainties". Included are a schematic representation of the deep learning algorithm (DLA), a comparison of the prediction of the DLA based on either demodulated beamformed (dmBF) image or modulated beamformed (mBF) image as input, the prediction from experimental data for a DLA trained with simulation data and the uncertainty estimation of the instances presented in the main text. The reconstruction of a simulated vessel-like object, provided by the DLA trained on the simulated leaf dataset, is also presented.

Keywords: Photoacoustic imaging; Deep learning; Visibility artefacts; Monte Carlo dropout; Bayesian neural network

1. Network architecture

Unet is a well known network architecture first developed for segmentation task. Our implementation is shown in Fig. S1. It is a convolutional neural network composed of two paths: the contracting and expanding path. The first one, called the encoder, is a traditional stack of convolutional and pooling layers where the network extracts more and more complex features. The second one, called the decoder, is the symmetric expanding path where

*Corresponding author: guillaume.godefroy@univ-grenoble-alpes.fr

pooling operations are replaced by upsampling operators to recover at the output the resolution of the input. Context information is propagated from the encoder to the decoder through skip connections to provide local information to the global information while upsampling (black arrows). The weights are initialized with samples from a truncated normal distribution centered on 0 with standard deviation depending of the number of units in the weight tensor. Dropout layers are added to this architecture. Dropout is a popular regularization technique to limit overfitting. A certain set of neurons, chosen randomly, are disabled at each training step. This prevents units from co-adapting too much and forces the network to learn more robust features. Batch normalization normalizes the output of the previous activation layer by subtracting the batch mean and dividing by the batch standard deviation. It helps to speed up the learning and also reduces overfitting by adding some noise, similarly as dropout.

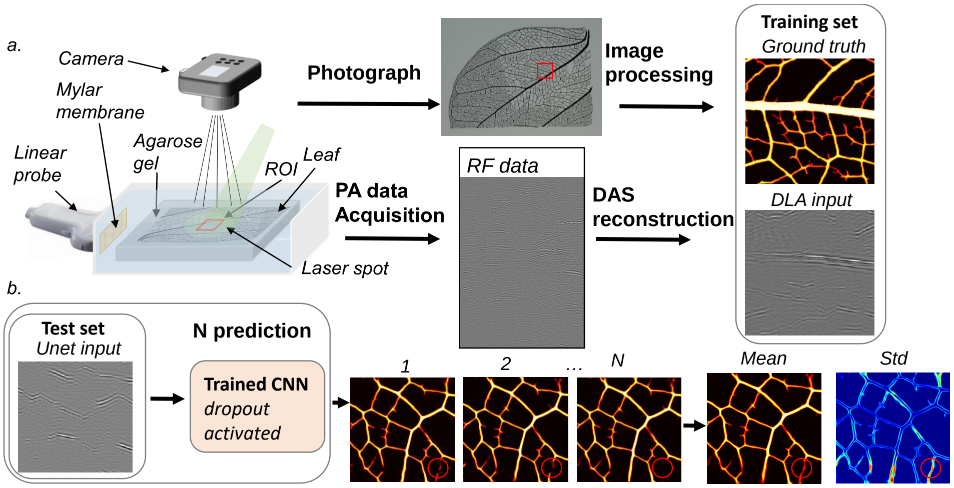

Figure S1: Network architecture.

2. mBF or dmBF image as input of the network

The input of the network is obtained from the delay and sum algorithm (DAS) applied to time signals. When applied to real time signals, DAS provides a mBF image. When applied to complex signals obtained with a Hilbert transform, DAS provides a complex image whose modulus is the dmBF image. The mBF image and the dmBF image are the two types of input that we

consider here. One DLA was trained for each type of input, the corresponding predictions are displayed in Fig. S2. The algorithm performs better on the mBF image, leading to a scaled and shifted structured similarity index (sSSIM) of 0.76 instead of 0.72. The prediction from the dmBF image suffers from more artefacts (arrows) and the DLA fails to recover the true vessels thicknesses, which are over estimated. The mBF image, despite being more different from the true physical structure of the object than the dmBF image, and thus from the ground truth, carries more information to be captured by the network.

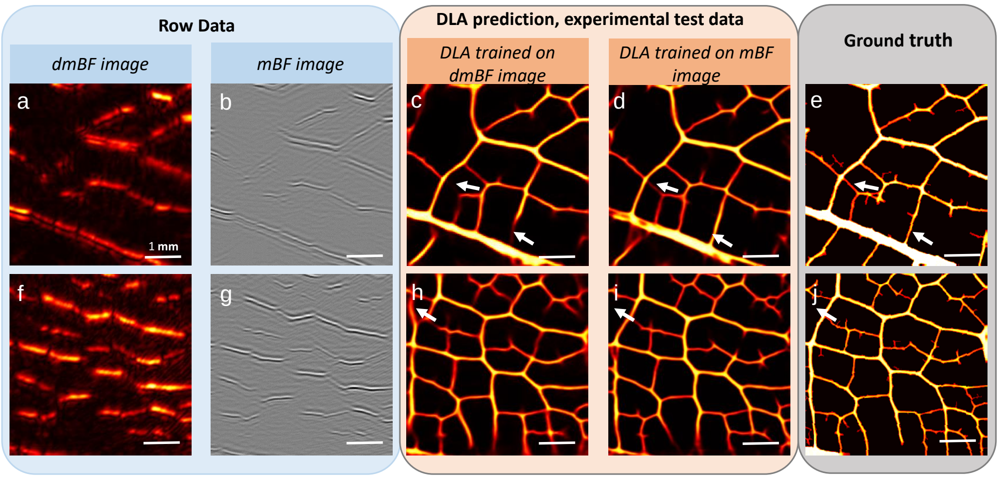

Figure S2: Deep learning algorithm (DLA) prediction on experimental data for dmBF PA image or mBF PA image as input, two examples.
a, f, dmBF image. **b, g,** mBF image. **c, h,** Prediction with DLA trained on dmBF data. **d, i,** Prediction on experimental data with DLA trained on mBF image. **e, j,** Ground truth.

3. Reconstruction of experimental data with DLA trained on simulation data

Predictions from neural networks trained with simulation datasets or experimental datasets are presented in Fig. S3. Although the DLA trained on simulation data still manages to find several vertical structures that are not visible on the DAS image, the predicted image is polluted by a lot of artefacts. Clearly, experimental data are necessary to train efficiently the model. However, as shown in the main text, pretraining the network on a simulation dataset allows reducing the size of the experimental training set.

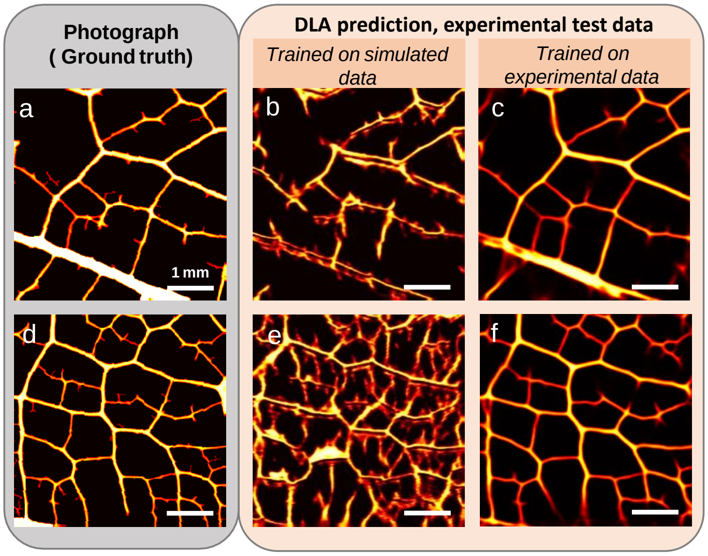

Figure S3: Deep learning algorithm (DLA) prediction on experimental data for DLA trained on simulated data and DLA trained on experimental data, two examples. **a, d,** Ground truth. **b, e,** Prediction with DLA trained on simulated data. **c, f,** Prediction on experimental data with model trained on experimental data.

4. Uncertainty estimation

Uncertainty estimation of the two previous examples are presented in Fig. S4. Similarly to the example in the main text, the standard deviation (std) map helps to locate errors in the reconstruction such as invented structures and incorrect orientation or position (arrows). One can notice that although the value of the std is higher at location of some missing veins (star), some of them are not even displayed on the std (circle). The lack of information in the data may be more important for these structures, misleading the DLA.

5. Generalization ability

This work was mostly dedicated to demonstrate the capacity of a neural network to correct visibility artefacts on a specific class of object. However the possibility of reconstructing objects from another class is an open question. To illustrate this problematic on a specific example, we used the network trained on leafs (simulated signals) to reconstruct the PA image of a vessel-like structure (test image from the k-wave package, http://www.k-wave.org/). The result is presented in Fig. S5. Although the class of the

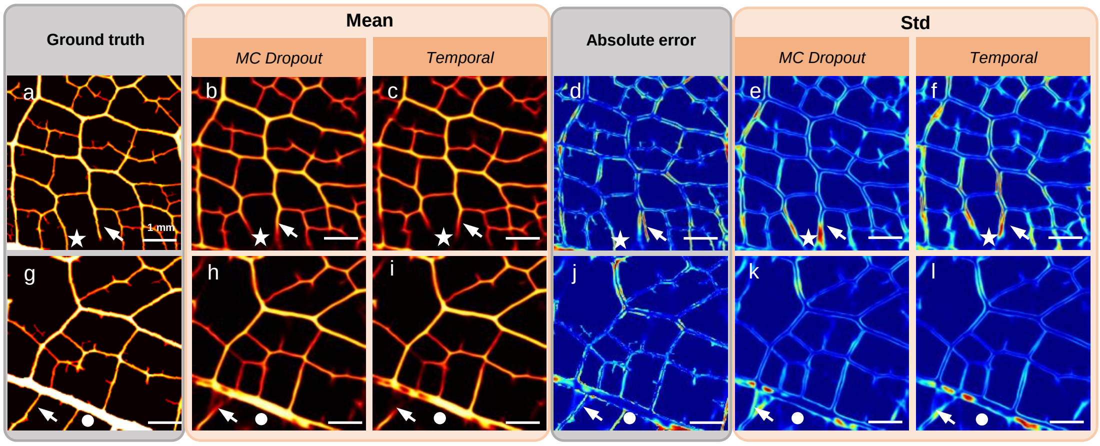

Figure S4: Uncertainty estimation on experimental mBF image. **a, g,** Ground truth: photograph of the object. **b, h,** Mean image of the object computed over 20 inferences generated from a unique acquisition with the deep learning algorithm, dropout activated. **e, k,** Corresponding STD. **c, i,** Mean image of the object computed over prediction generated from several RF signals acquired at different time with the deep learning algorithm, dropout disabled. **f, l,** Corresponding STD **d, j,** Absolute error between the ground truth (a,g) and the mean (b,h).

object is clearly different, the algorithm turns out to perform extremely well on this specific example, as visibility artefacts are removed, suggesting no or low overfitting. However, this is just one example, obtained from simulated data, and further studies are required to investigate the generalization limits of the network, especially for experimental data.

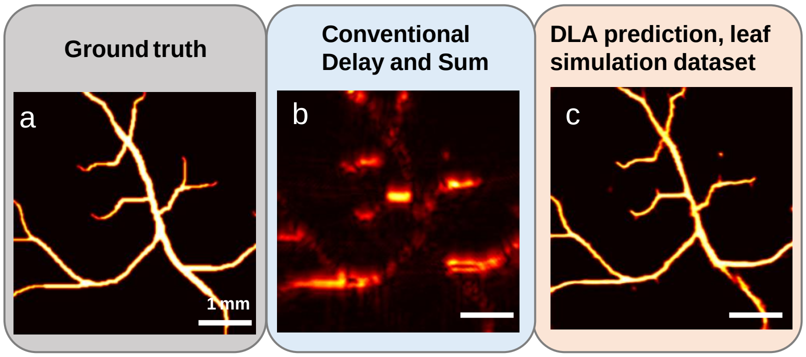

Figure S5: Reconstruction of a simulated vessel-like objet with a network trained on simulation from the leaf object. **a,** Ground truth. **b,** dmBF image, delay and sum. **c,** Prediction of the deep learning algorithm.
